# Identification of Human Semiochemicals Attractive to the Major Vectors of Onchocerciasis

**DOI:** 10.1371/journal.pntd.0003450

**Published:** 2015-01-08

**Authors:** Ryan M. Young, Nathan D. Burkett-Cadena, Tommy W. McGaha, Mario A. Rodriguez-Perez, Laurent D. Toé, Monsuru A. Adeleke, Moussa Sanfo, Traore Soungalo, Charles R. Katholi, Raymond Noblet, Henry Fadamiro, Jose L. Torres-Estrada, Mario C. Salinas-Carmona, Bill Baker, Thomas R. Unnasch, Eddie W. Cupp

**Affiliations:** 1 Center for Drug Discovery and Innovation, University of South Florida, Tampa, Florida, United States of America; 2 Global Health Infectious Disease Research Program, Department of Global Health, University of South Florida, Tampa, Florida, United States of America; 3 Centro de Biotecnología Genómica, Instituto Politécnico Nacional, Reynosa, Tamaulipas, México; 4 Facultad de Medicina, Universidad Autónoma de Nuevo León, Monterrey, Nuevo León, México; 5 African Programme for Onchocerciasis Control, Ouagadougou, Burkina Faso; 6 Department of Biological Sciences, Osun State University, Osogbo, Nigeria; 7 Programme National de lutte contre l'onchocercose, Direction de la lute contre la maladie, Ministère de la Santé, Ouagadougou, Burkina Faso; 8 Department of Biostatistics, School of Public Health, University of Alabama at Birmingham, Birmingham, Alabama, United States of America; 9 Department of Entomology, University of Georgia, Athens, Georgia, United States of America; 10 Department of Entomology and Plant Pathology, Auburn University, Auburn, Alabama, United States of America; 11 Centro Regional de Investigación en Salud Pública, Instituto Nacional de Salud Pública, Tapachula, Chiapas, México; National Institute of Allergy and Infectious Diseases, United States of America

## Abstract

**Background:**

Entomological indicators are considered key metrics to document the interruption of transmission of *Onchocerca volvulus*, the etiological agent of human onchocerciasis. Human landing collection is the standard employed for collection of the vectors for this parasite. Recent studies reported the development of traps that have the potential for replacing humans for surveillance of *O. volvulus* in the vector population. However, the key chemical components of human odor that are attractive to vector black flies have not been identified.

**Methodology/Principal Findings:**

Human sweat compounds were analyzed using GC-MS analysis and compounds common to three individuals identified. These common compounds, with others previously identified as attractive to other hematophagous arthropods were evaluated for their ability to stimulate and attract the major onchocerciasis vectors in Africa (*Simulium damnosum sensu lato*) and Latin America (*Simulium ochraceum s. l.*) using electroantennography and a Y tube binary choice assay. Medium chain length carboxylic acids and aldehydes were neurostimulatory for *S. damnosum s.l.* while *S. ochraceum s.l*. was stimulated by short chain aliphatic alcohols and aldehydes. Both species were attracted to ammonium bicarbonate and acetophenone. The compounds were shown to be attractive to the relevant vector species in field studies, when incorporated into a formulation that permitted a continuous release of the compound over time and used in concert with previously developed trap platforms.

**Conclusions/Significance:**

The identification of compounds attractive to the major vectors of *O*. *volvulus* will permit the development of optimized traps. Such traps may replace the use of human vector collectors for monitoring the effectiveness of onchocerciasis elimination programs and could find use as a contributing component in an integrated vector control/drug program aimed at eliminating river blindness in Africa.

## Author Summary

Human landing collections, which are the current standard for collecting the black fly vectors of *Onchocerca volvulus*, the causative agent of river blindness, are inefficient and pose certain ethical issues. As entomological methods are among the primary techniques recommended by the international community for verifying the elimination of onchocerciasis, there is a need to develop alternative methods to collect these vectors. Recent studies have demonstrated that traps baited with CO_2_ and dirty clothing have the potential to replace human landing collections for this purpose. However, for these traps to be widely applied, it will be necessary to develop a consistent bait formulation. To this end, volatile compounds from human sweat that attract the principal black fly vectors of *O. volvulus* in Africa and the Americas have been identified and used to optimize traps that specifically collect these insects. To achieve this milestone, we report the use of electroantennography and behavioral assays to identify human compounds that are neurostimulatory to these vectors, and demonstrate that these compounds are attractive to the vectors in field studies using previously developed trap platforms. The development of such a defined bait formulation will permit the widespread use of these traps by onchocerciasis elimination programs in Africa and the Americas.

## Introduction

*Onchocerca volvulus*, the etiological agent of “river blindness”, remains a major public health threat in much of Africa and in a cross-border focus between Venezuela and Brazil [1]. Recent studies suggest that mass distribution of Mectizan (ivermectin) may be effective in achieving local elimination of the parasite [2], [3] and have resulted in a strategic shift from control of river blindness towards elimination [1]. The World Health Organization and other international onchocerciasis elimination programs rely on measurement of the presence and intensity of transmission of the parasite for the verification of onchocerciasis elimination, both of which are measured by surveillance of the vector population [4].

Current methods to measure parasite transmission employ human landing collections to attract and collect vector black flies (*Simulium* spp.); infection data from the flies collected are then used for real-time surveillance, implementation of mass drug treatment and decision-making based on the outcome of epidemiological models [5]. However, the use of such collections is problematic for several reasons. First, collections of this type pose ethical questions in endemic areas, as they might result in exposure of the collectors to *O. volvulus* and perhaps other uncharacterized pathogens present in these flies. Second, verifying interruption of transmission requires collecting and screening large numbers of flies. For example, the current guidelines adopted by the Onchocerciasis Elimination Program for the Americas require that sufficient flies be collected from each endemic community to ensure that the upper bound of the 95% confidence interval of the number of flies carrying the infective stage of the parasite (L_3_) is less than 0.05%, or 1/2000 flies [5]. To achieve this, it is necessary to collect and screen at least 6000 flies from each community [6], [7]. Human landing collections are labor intensive and often cannot collect such a large number of flies in a cost effective manner. For these reasons, there is a need to develop an alternative to human landing collections for onchocerciasis surveillance.

Recent efforts have been made to develop traps capable of collecting vector black flies in a variety of ecological settings [8], [9]. These studies have shown that a novel trap platform, the Esperanza Window Trap, which consists of an adhesive-coated blue or black and blue striped fabric square, when baited with CO_2_ and worn clothing collected numbers of vector black flies that were similar those obtained by human landing collections [8], [9]. Both CO_2_ and the odor baits were found to be necessary to attract significant numbers of vector flies [8], [9]. However, to be widely applied as a surveillance tool to replace human landing collections, it will be necessary to develop a consistent bait formulation to replace the worn clothing as bait, allowing the traps to function consistently over time and space. In the experiments presented below, we report the results of studies identifying human-derived compounds that are attractive to *Simulium ochraceum sensu lato* and *S. damnosum s. l.* The savanna dwelling sibling species of *Simulium damnosum s.l.* are the vectors of the blinding form of *O. volvulus* and represent the most important vector species on the African continent, while *S*. *ochraceum s.l.* was the primary vector in the historically largest endemic foci in Mexico and Guatemala [10]. To accomplish this goal we have utilized an approach involving a combination of electrophysiological and behavioral assays, a process that has proven to be effective for the identification of attractive volatiles for a variety of other medically important vector species [11]–[13].

## Materials and Methods

**Figure 1:**
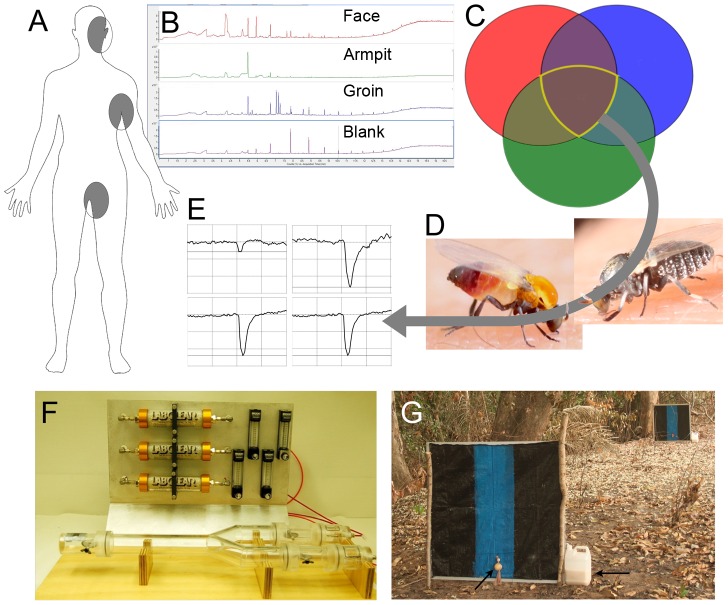
Strategic plan of the study. A. Bodily locations of sweat collection from three volunteers. B. Gas chromatogram patterns of sweat components. C. Venn diagram analysis of compounds common among the three volunteers. D. Electroantennography of *Simulium ochraceum s.l.* (left) and *S. damnosum s.l* (right). E. Representative action potentials from *S. ochraceum s.l.* and *S. damnosum s.l*. antennae. F. The Y tube apparatus. Flies were introduced into the release chamber (left) that opens to stimulus arms ending in trap chambers. The flow rate of activated carbon purified air was 1.5L/min. G. Esperanza Window Traps stationed in the field in Bodajugu, Burkina Faso. Left arrow indicates aroma beads baited with candidate compounds suspended in a nylon stocking. Right arrow indicates the container emitting organically derived CO_2_.

The approach to identifying human kairomones that were attractive to the major vector species employed a systematic methodology that began with chemical analysis of sweat samples in the laboratory and ended in evaluation of candidate compounds in the field in Mexico and Burkina Faso. An overview of each step in this progression is illustrated in Fig. 1.

### Collection and analysis of sweat samples

Sweat was collected from the face, armpit and groin areas of three male volunteers after each subject engaged in 30 minutes of aerobic exercise (Fig. 1A). These areas were chosen as *S. ochraceum* s.l. usually bites on the head and upper part of the body [14] while *S. damnosum* s.l. targets the lower part of the body [15], and previous studies demonstrated that sweat impregnated shirts were attractive to *S. ochraceum* s.l. [8] while sweat impregnated pants were attractive to *S. damnosum* s.l. [9]. Samples were taken by wiping the chosen areas thoroughly with sterile cotton swab. Samples were collected from each individual on three separate occasions over a three-week period. The cotton wool containing each sweat sample was immediately placed into a separate headspace-analysis vial and sealed. Starting immediately, then every 24 hours thereafter for 5 days, headspace analysis of each sweat sample was conducted by Gas Chromatography (GC) using carboxen-polymethylsiloxane and polyacrylate solid phase microextraction (SPME) fibers (Fig. 1B). Samples were analyzed sequentially over a five day period to permit the development of metabolites from the skin flora, as these have been shown to be attractive in previous studies [16], [17]. Samples were maintained at ambient temperature between analyses. Prior to introduction of an SPME fiber for each analysis, the sweat sample was incubated at 50°C for three minutes. The SPME fiber was left in contact with the headspace for five minutes before being removed for GC analysis. Control samples, comprising the full experimental sample preparation method but without application of the cotton wool to the human subjects, were analyzed separately.

SPME fibers were analyzed on an Agilent 7200 GC-QToF. The sweat components were thermally desorbed in the GC inlet at 320°C, where the individual components were resolved on a HP-5ms capillary column (30 m, i.d. 0.25 mm) using helium as the carrier gas at a flow rate of 1.2 mL/min. The column was heated from 60°C to a final temperature of 320°C at a rate of 20°C/min. Compounds were ionized using electron ionization (EI) for initial identification. Chemical ionization (CI) was used to confirm molecular ions using methane as the reagent gas and both positive and negative mode (40–110 eV, compound dependent) to obtain optimum signal-to-noise ratio.

The total ion chromatographs were deconvoluted using Agilent's Deconvolution Algorithm and the resulting EI induced fragmentation patterns were exported as CEF files and imported into Mass Profiler Professional for statistical analysis. Datasets, prepared for each time point and from both SPME fiber materials, were combined to create an entity list for each subject containing all compounds present in their sweat. Compounds in the control samples (i.e. those not containing and sweat) were removed from the entity lists. Spectra of entities identified as common in the sweat of all individuals were compared to the fragmentation patterns contained in the NIST/Wiley 2011–2012 mass spectra library for a tentative identification. Finally, retention times, molecular ions and fragmentation patterns of commercially available standards were compared to tentatively identified compounds for final confirmation.

### Electroantennogram (EAG) procedures

Host-seeking *Simulium* spp. females were captured by vector collectors in the field near Bodadougou, Burkina Faso (*S. damnosum sensu stricto* and *S. sirbanum*) and Unión Juárez, Chiapas, México (*S*. *ochraceum sensu lato*), identified using morphological characters, and used for experiments in a field laboratory within 24 hours of capture (Fig. 1D). Preliminary trials indicated that a preparation consisting of the thorax and head produced the best signal to noise ratio for EAG recordings. Glass electrodes (0.2 mm dia. silver chloride-coated wires) were connected to the tip of one antenna and the exposed internal tissues of the metathorax. All test compounds were purchased from Sigma Aldrich or Fisher Scientific; only compounds with purity higher than 98% (with the exception of lactic acid which was 90% pure) were used. Unless specified, all chiral molecules were racemic mixtures of enantiomers. Test compounds were dissolved in hexane (HPLC grade, ≥95%) or distilled water to yield a solution (1/100, or roughly 80 mM) for EAG analysis.

Test solutions (10 µL) were applied to a strip of Whatman filter paper (Whatman, Inc. USA), the solvent allowed to evaporate, and the paper strip inserted into a glass Pasteur pipette. An air stimulus controller (CS-55, Syntech, the Netherlands) generated a pulse (0.2 s duration) of filtered air that introduced headspace volatiles from the pipette into a continuous stream of humidified air (1000 mL/min) that was directed at the antenna of the test insect, initiating the EAG recording. Electrodes used to monitor a stimulus were Ag/AgCl wire submerged in freshly prepared saline solution (750 mg NaCl, 35 mg KCl and 29 mg CaCl_2_•2H_2_O in 100 mL of distilled water), which was placed in each of the glass electrodes prior to analysis and changed between flies. Individual compounds were randomly assigned to one of 10 groups (5–7 compounds per group). Within each group, the order in which compounds were assayed was randomized for each trial. EAG responses of hexane (control) and a blank (puff of air) were recorded before and after each group of compounds (Fig. 1E). Each compound was initially assayed in three replicates via EAG. Compounds that elicited an EAG response that did not differ from the control (hexane) response were not used in subsequent assays. Compounds that elicited EAG responses consistently greater than hexane in 2–3 replicates were further assayed with additional replicates.

EAG responses of compounds were normalized to the preceding response of the control stimulus (hexane). A t-test was used to determine if normalized EAG responses of compounds differed from that of the control (α = 0.05). Compounds eliciting significant EAG responses then became candidates for Y-tube evaluation.

### Y-Tube assays

Wild-caught *Simulium* spp. were collected as noted above, stored in plastic vials, and transported in coolers to a local field laboratory. Experiments were conducted within 24 hours of collection. Behavioral bioassays were conducted in a Y-tube olfactometer (Fig. 1F), modified from an earlier design [18]. The Y-tube was made of transparent acrylic with an internal diameter of 1.5 inches and consisted of four sections: release chamber, Y-split chamber, stimulus arm trap chambers, and stimulus chambers. The release chamber contained an aspirator entry point on the ventral side, a fabric mesh screen on the dorsal side, and a metal-mesh rotating door in the apical side. The mesh partitions allowed air to flow through the release chamber and the rotating door was used to release the flies after acclimation into the Y-split chamber, which connected the release chamber to the stimulus arm trap chambers. Each stimulus arm trap chamber contained a rotating metal mesh screen door, which closed after the test run to prevent any flies from entering or exiting the stimulus arm chamber. A stimulus chamber was connected to the apical end of the stimulus arm trap chamber that was separated by a fabric mesh screen. The stimulus chamber was accessible by a sliding door. During all experiments, a black cloth covered the Y-tube olfactometer completely, except the apical end of the stimulus chambers. This opening allowed light (Utilitech fluorescent plant grow light) to enter the apical end of the stimulus chambers and was required to initiate activation of the flies. Hydrocarbon-filtered air (LabClear, Diamond Tool and Die, Inc., Oakland, CA) was supplied into the apical end of the stimulus chambers by two separate air-lines from an air pump (Greentrees Hydroponics, Vista, CA). The flow of filtered air into the stimulus chambers was regulated (Brooks Instruments, Hatfield, PA) at a rate of 1.5 liters per minute.

Initially, each group of flies (n = 20) was acclimated to filtered air pumped through the Y-tube while held in the release chamber for 10 minutes, with no compound present in either arm. After acclimation, the flies were released and allowed 1 minute to make a choice in the Y-tube. If no preference to a particular arm was observed (*i.e.* p>0.05 using the Chi Square likelihood ratio test based on the multinomial model described below), the flies were collected back into the release chamber. If a preference was observed in a particular control run, the flies were discarded and the Y-tube was cleaned and the process repeated until no preference was observed. A test run was then performed in which one arm contained the test compound and the other arm the solvent alone. Regardless of whether attraction to a given compound was observed, the Y-tube was disassembled and cleaned with a detergent water solution and ethanol after every other experiment (i.e. after the paired control and test runs). A total of six replicates per compound were performed, assaying a total of 120 flies per compound.

Liquid test and control stimuli were introduced into the Y-tube olfactometer by impregnating 20 µl of the test or control solution onto a 2.5-cm circular piece of filter paper (Whatman grade 1, GE Healthcare, Little Chalfont, UK). Sufficient time was allowed for the solvent to evaporate before the impregnated filter paper was placed in the stimulus chamber, where a clamp held the filter paper in place. Each filter paper was prewashed with hexane before the experiment. The attraction response was measured for each test run replicate and all compounds were tested using ten-fold dilutions (1∶10 v/v; 1∶100 v/v; 1∶1000 v/v) to determine the overall range of attractiveness of each candidate compound. The test attraction response was calculated using the formula: Test attraction response  =  (*T* x 100)/(*T*+*C*) where *T* denotes the number of flies trapped on the test stimulus side and *C* denotes the number of flies trapped on the control stimulus side. The same calculation was performed to find the control attraction response. The proportion of the attraction responses were transformed via arcsine transformation before the means were calculated for comparison. The proportions of flies that chose the control and the test stimuli were compared using a likelihood ratio test based on a multinomial probability model. Test attraction responses for all test stimuli were compared by means of pair-wise comparisons based on the multinomial model with alpha level adjusted to account for multiple comparisons, using a custom program written in FORTRAN 95. The program is available upon request.

### Preparation of continuous-release baits and kinetic analysis of compound release

Compounds found to be attractive in the Y tube assay were absorbed into plastic aroma beads (Bitter Creek Candle Supply [www.candlesupply.com]) for use as artificial baits. All beads were prepared at a ratio of 0.222 mL active compound per 1 g of aroma beads, and allowed to absorb candidate compounds over a 12-hour period. The beads were then allowed to rest for 24 hours to permit residual unincorporated solution to evaporate. To determine kinetics of compound release, two beads loaded with each compound were then placed into individual headspace vials for analysis, and incubated at room temperature. The amount of compound remaining was quantified by GC/MS at 24-hour intervals for five days. Samples were introduced into the GC inlet after being allowed to adsorb onto a carboxen-polymethylsiloxane SPME fiber from a headspace vial incubated at 35°C for 2 minutes. SPME fibers were analyzed on an Agilent 7000 GC/QqQ MS. The individual compounds were thermally desorbed in the GC inlet at 320°C, where the retention time was monitored on a HP-5ms capillary column (30 m, i.d. 0.25 mm) using helium as the carrier gas at a flow rate of 1.2 mL/min. The column was heated from 30°C to a final temperature of 320°C at a rate of 40°C/min. Compounds were ionized using electron ionization (EI) where the total ion chromatogram was integrated and used to determine the percentage of compound remaining, using Agilent's MassHunter B.5.00 software.

### Field evaluation of baits

Beads containing the attractive compounds were prepared as described above. The field-ready bait was prepared by placing aroma beads loaded with individual compounds (4.5 g per compound) into nylon stockings, along with 4.5 g of powdered ammonium bicarbonate separated from the beads by a knot in the stocking. The baits were placed on each side of an EWT trap, utilizing versions of the trap that were optimized for use in Mexico [8] and Burkina Faso [9]. As previous studies had demonstrated that CO_2_ was necessary to induce attraction to the trap platforms baited with used clothing [9] all traps were also baited with organic CO_2_, which was prepared as previously described [8]. Comparisons were made between traps with CO_2_ alone (control) and traps with CO_2_ + the mixture of odorant compounds (Fig. 1G).

For *S*. *ochraceum s.l.*, traps were set in San José, Chiapas, México, and run from 8 am to noon for 6 days. The field evaluation was conducted during the early dry season; January 16^th^ through February 1^st^ 2014. The mixture of odorant compounds evaluated for the collection of *S. ochraceum s.l.* contained 1-octen-3-ol, 1-octanol, acetophenone, hexanal, and ammonium bicarbonate. Seven pairs of traps, located 15-20 meters apart, were used to evaluate each condition and the position of each trap was rotated daily to avoid location bias. *Simulium damnosum s.l.* data were collected using paired traps run at Bodajugu, Burkina Faso, over a seven-day period. Traps were run from sunrise to sunset, and their positions alternated from day to day. The trap evaluation was conducted in the dry season (March 27^th^ through April 1^st^). This portion of the river is located downstream of a dam, resulting in conditions that support fly breeding throughout the year. Baits evaluated for the collection of *S. damnosum s.l.* contained hexanoic acid, heptanoic acid, octanoic acid, nonanoic acid, 1-decanal, acetophenone, and ammonium bicarbonate. Statistical analysis of data collected in both field settings was performed using SAS Proc GENMOD to analyze the data as a Negative Binomial regression.

### Ethical clearance

The experiments included in this study were reviewed and approved by the Institutional Review Board for Human Subjects Research of the University of South Florida. The board declared that the project qualified for the expedited review procedure authorized by federal regulations 45CFR46.110 and 21 CFR 56.110 under category 3: “Prospective collection of biological specimens for research purposes by noninvasive means”. The review board further determined that the work qualified for a waiver for documentation of informed consent as outlined in federal regulations at 45CFR46.117(c), which state that an IRB may waive the requirement for the investigator to obtain a signed consent form for some or all subjects. As a result, oral consent was obtained from all participants. Participants were asked to sign a form indicating that the purpose and procedures employed in the study had been explained to them. The signed forms were retained by the principal investigator of the project.

## Results

**Figure 2:**
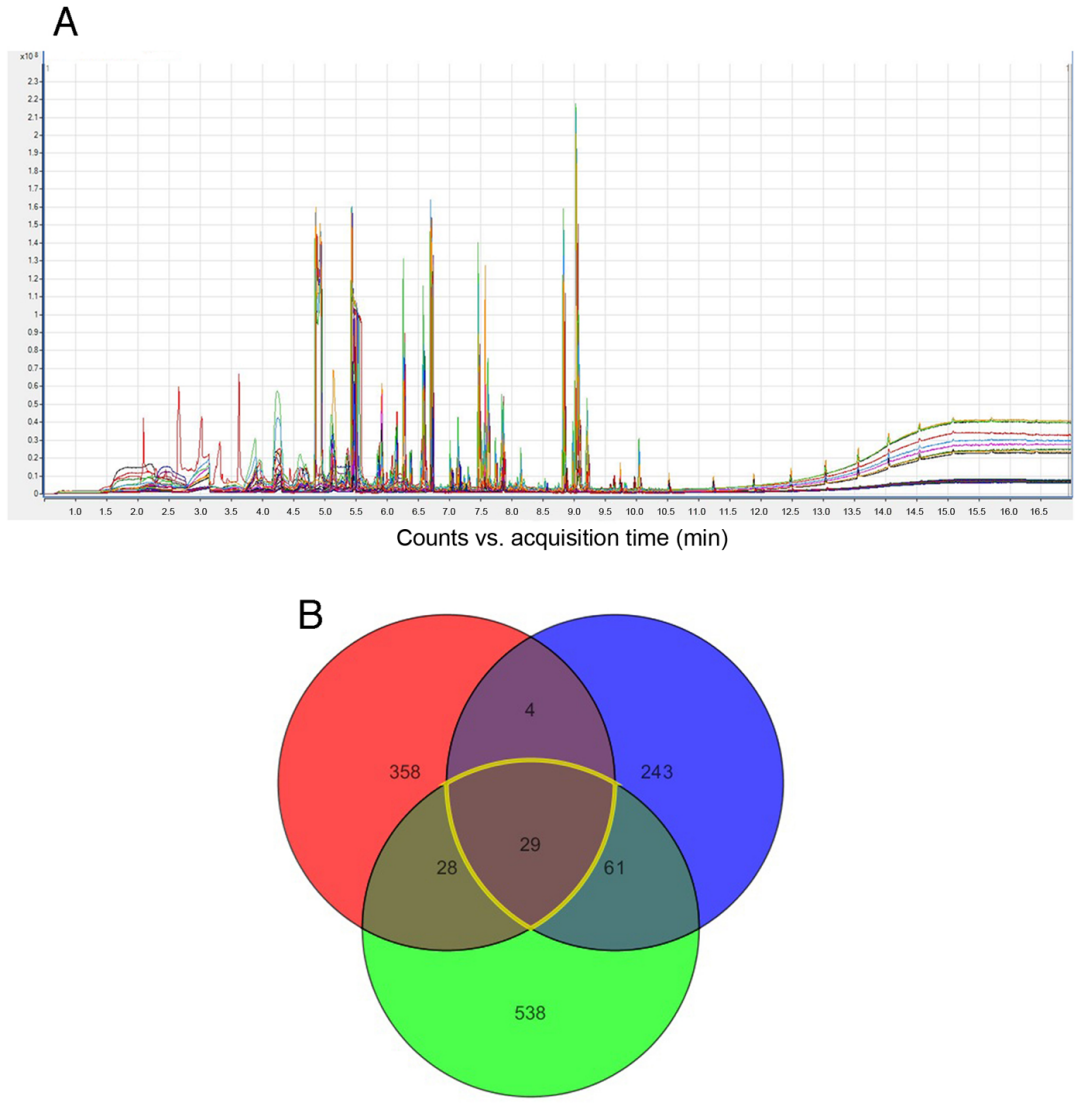
Identification of human sweat compounds. Panel A: The overlaid total ion chromatograms from once daily sampling of human armpit sweat of a single Caucasian male over five days. Each sample was run in triplicate; thus 15 traces are visible in the figure. Panel B: Venn diagram of the compounds found to be unique or shared among the three individuals enrolled in the study.

As a first step in identifying human odorants attractive to *Simulium spp*., sweat compounds were identified from samples collected from three individuals using GC-MS as described in Materials and Methods (Fig. 2, Panel A). A total of 1,261 compounds were identified from the three volunteers, of which the vast majority (90%) were unique to one individual (Fig. 2, Panel B). Just 29 compounds were common to all three individuals. These were chosen for further evaluation in the electrophysiological and behavioral assays. In addition, 25 other compounds consistently reported in the literature to occur in sweat and/or eliciting attractive behavior in other blood-feeding arthropods [19], [20] were included in the subsequent studies. Taken together, 54 compounds were evaluated by electroantennography (Table 1).

**Table 1:** Putative kairomones selected for EAG study of *Simulium spp*.

Class of Compound	Compound Name	Identified from [reference]	Attractive to [reference]
Carboxylic Acids	Heptanoic acid*	Groin (human)	–
	Lactic acid*	Arms & Armpit (human) [19]	*Anopheles gambiae* [19]
	Octanoic acid	Sweat & Feet (human) [20], [38], [51]	–
	Hexanoic acid	Sweat, Feet (human) & Rumen (bovine) [30], [38], [45], [51]	*Stomoxys calcitrans, An. gambiae* [30], [45]
	Nonanoic acid*	Sweat, Skin & Groin (human) [20], [38]	–
	Isobutyric acid	Sweat, Feet (human) & Rumen (bovine), [20], [45], [51]	*S. calcitrans* [20], [45], [51]
	4-Methoxybenzoic acid*	Armpits (human)	–
	DL-Serine	Armpits & forehead (human) [52]	–
	Tetradecanoic acid	Sweat & Skin (human) [20], [38]	–
	Octadecanoic acid	Sweat (human) [20]	–
	Hexadecanoic acid	Sweat & Skin (human) [20], [38]	–
	Adipic acid	Skin (human) [38]	–
	Isophthalic acid*	Armpits (human)	–
	Isovaleric acid	Feet (human) & Rumen (bovine) [45], [51]	*S. calcitrans* [45], [51]
	Propionic acid	Feet (human) & Rumen (bovine) [30], [45], [51]	*S. calcitrans, An. gambiae* [30], [45], [51]
	Butyric acid	Sweat, Feet (human) & Rumen (bovine) [30], [45], [51]	*S. calcitrans, An. gambiae* [30], [45], [51]
	Pentadecanoic acid	Sweat (human) [20], [38]	–
	Decanoic acid	Sweat & Feet (human) [20], [38], [51]	–
	Undecanoic acid*	Armpits (human)	–
	Tridecanoic acid	Sweat & Skin (human) [20], [38]	–
	Linoleic Acid*	Armpits & Groin (human)	–
	2-Methylhexanoic acid*	Armpits (human)	–
	Oleic Acid	Sweat (human) [20]	–
Alcohols	Tetrahydrofurfuryl alcohol	Skin (human) [38]	–
	3-Octanol	Rumen (human), [45], [53]	*S. calcitrans* [45]
	1-Octen-3-ol	Feet (human), Rumen (bovine) & Fungal [45], [51], [53]	*S. calcitrans* [45]
	2-Ethyl-1-hexanol	Rumen (bovine) [45]	*S. calcitrans* [45]
	*cis*-3-Hexen-1-ol	Rumen (bovine) [45]	*S. calcitrans* [45]
	1-Octanol	Rumen (bovine) [45]	*S. calcitrans* [45]
	1-Decanol*	Groin (human)	–
	1-Heptadecanol*	Armpits (human)	–
	1-Pentadecanol*	Armpits & Groin (human)	–
	1-Tetradecanol*	Groin (human)	–
	2,4,4-Trimethyl-1-pentanol*	Armpits (human)	–
	1-Octadecanol*	Armpits (human)	–
Aldehydes	Nonanal*	Armpits, (human) bovine & rabbit [36], [38], [52]	*Amblyomma variegatum, Culex quinquefasciatus* [52] [36]
	Hexanal*	Armpits (human) bovine & rabbit [38], [52]	*Am. variegatum* [52]
	Tetrahydro-2-furancarboxaldehyde*	Skin (human), bovine & rabbit [38], [52]	*Am. variegatum* [52]
	Decanal*	Armpits, Forearm (human) & Rumen (bovine) [30], [36], [38], [45]	*Stomoxys calcitrans*, *Cx. quinquefasciatus, An. gambiae* [30], [36], [45]
	1-Pentadecanal	Armpits (human)	–
Alkanes	Pentadecane*	Groin (human)	–
	Undecane	Breath (human) [43]	–
	Heptadecane	Skin (human) [38]	–
	Hexadecane*	Armpits & Groin (human)	–
Ketones	(+/-)-Dihydrocarvone	Rumen (bovine) [45]	*S. calcitrans* [45]
	Methyl acetoacetate*	Armpits (human)	–
	6-Methyl-3-hepten-2-one*	Sweat (human) & Rumen (bovine) [30], [45]	*S. calcitrans, An. gambiae* [30], [45]
	6,10-Dimethyl-5,9-undecadien-2-one	Skin (human) [38]	–
	Sodium pyruvate	Armpits & Forehead (human) [52]	–
	4-Methoxy-2H-chromen-2-one*	Armpits (human)	–
	Acetophenone	Rumen (bovine) Breath (human) [45] [43], [12]	*S. calcitrans* [43], [45] [12]
Others	3,6-Dimethylphthalic anhydride*	Armpits (human)	–
	3-Methyl indole*	Groin (human)	–
	*R*-(+)-Limonene	Rumen (bovine) & Breath (human) [43], [45]	*S. calcitrans* [43], [45]
	Cedryl acetate*	Armpits (human)	–
	Urea* (ammonia)	Armpits & Forehead (human) [54]	*Aedes aegypti* [48]

* Compound common to all individuals examined in this study.

**Figure 3:**
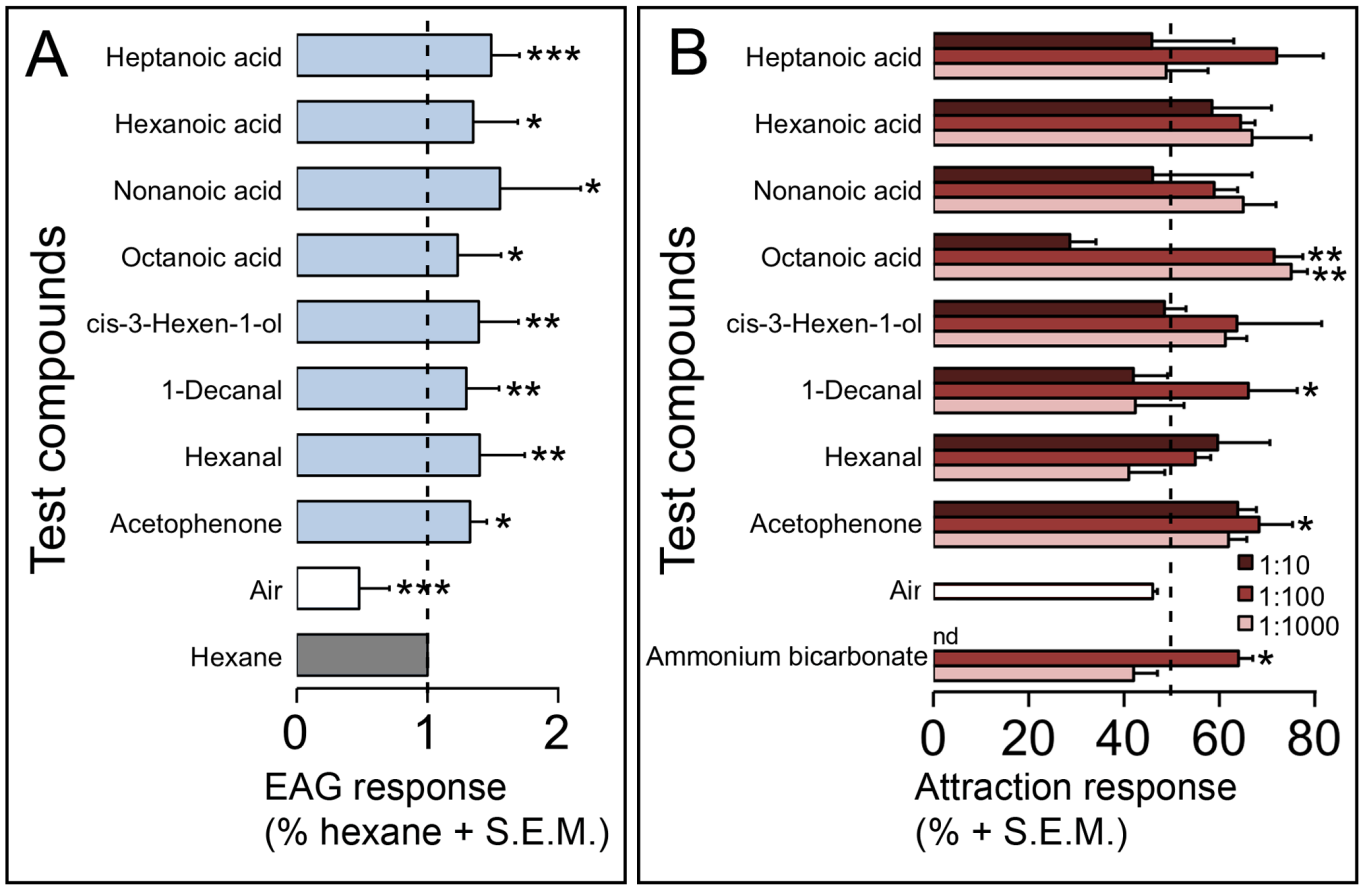
Identification of compounds attractive to *S. damnosum s.l*. Panel A: Electroantennogram (EAG) recording of responses to human sweat compounds. Average EAG response (+ SEM), relative to hexane). Only compounds inducing a significant EAG response are shown. Panel B: Y tube olfactometer assays of compounds found to be stimulatory in the EAG assay. Olfactometer controls  =  air and hexane. Error bars indicate the SEM and the column the mean percentage of flies present in the stimulus arm of the olfactometer at the end of the experiment. In each panel, * p<0.05, ** p<0.005, and *** p<0.0005; N = 3–5.

**Figure 4:**
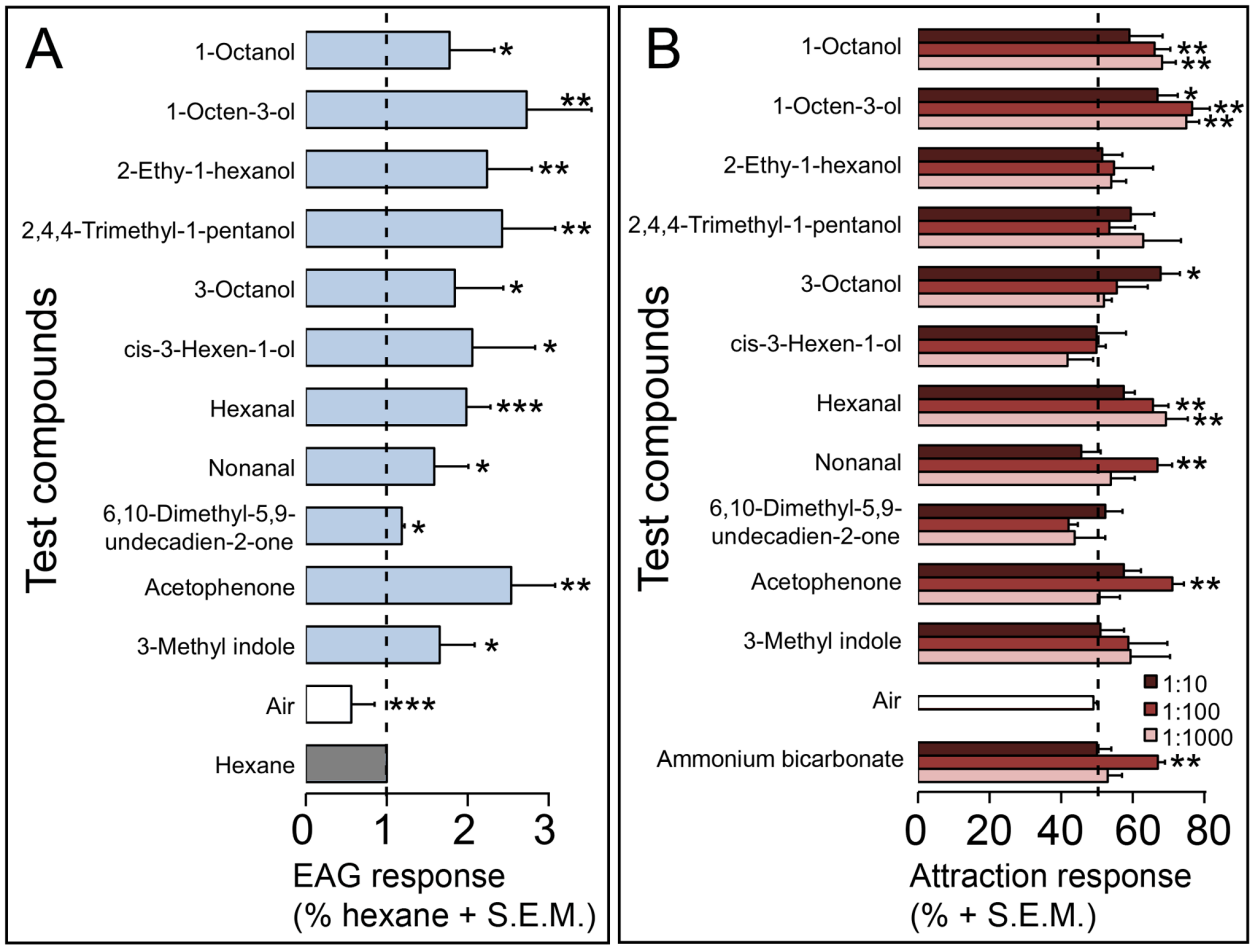
Identification of compounds attractive to *S. ochraceum s.l.* Panel A: electroantennogram (EAG) responses to human sweat compounds. Bars represent the average EAG response (+ SEM), relative to hexane). Only compounds inducing a significant EAG response are shown. Panel B: Y tube olfactometer assays of compounds found to be stimulatory in the EAG. Bars indicate the mean percentage of flies (+ SEM) present in the stimulus arm of the olfactometer at the end of the experiment. In each panel, * p<0.05, ** p<0.005, and *** p<0.0005; N = 3–5.

Putative soluble attractive compounds were then assayed for neurostimulatory effects via electroantennography (EAG) using field-collected, host-seeking flies. Of the 54 compounds tested, nineteen were EAG-active in one or both of the vector species (Fig. 3, Panel A and Fig. 4, Panel A). Differences were noted in the types of compounds attractive to each species. Several short chain carboxylic acids were stimulatory to *S. damnosum s.l.*, (Fig. 3, Panel A) while short chain aliphatic alcohols proved to be stimulatory to *S. ochraceum s.l*. (Fig. 4, Panel A). Three compounds were found to be neurostimulatory for both species (acetophenone, hexanal and *cis*-3-hexen-1-ol).

The EAG experiments identified a suite of compounds that were neurostimulatory to both vector species, but could not evaluate what type of behavioral response, if any, these stimulatory compounds might elicit. To answer this question, the neurostimulatory compounds were evaluated in a series of choice experiments using a Y-tube olfactometer, to identify which compounds might promote host-seeking behavior in nature [21]. Hexane solutions of octanoic acid (1∶100; 1∶1,000), decanal (1∶100) and acetophenone (1∶100) were found to be significantly attractive to *S*. *damnosum s.l.* (Fig. 3, Panel B). Other short chain carboxylic acids (e.g. hexanoic acid, heptanoic acid and nonanoic acid) were also found to elicit an attractive response, though these did not reach statistical significance (Fig. 3, Panel B). Seven compounds were attractive to *S. ochraceum s.l.* (Fig. 4, Panel B). These included all dilutions of 1-octen-3-ol, acetophenone (1∶100), 3-octanol (1∶10), nonanal (1∶100), 1-octanol (1∶100, 1∶1000) and hexanal (1∶100, 1∶1,000). Ammonium bicarbonate (1∶100 w/v), a solid compound not evaluated in the EAG screen for technical reasons, was also attractive to both species in the Y-tube assay (Fig. 3 and 4, Panels B).

**Figure 5:**
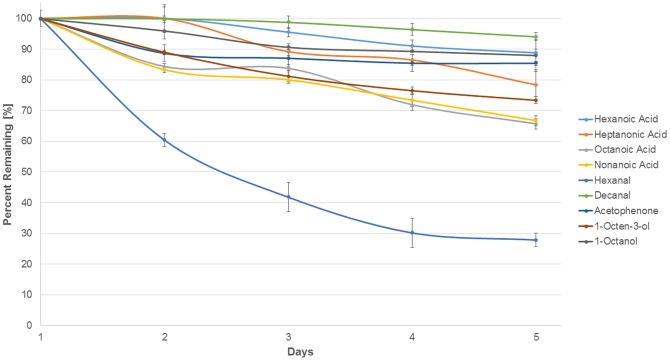
Release of attractive compounds from impregnated aroma beads. Beads were saturated with individual attractive compounds and the release of the absorbed compound measured over 5 days, as described in Materials and Methods. Error bars represent the standard deviation from 3 individual trials.

To verify that the compounds identified in the Y tube assay were also attractive in nature, slow-release baits of these compounds were prepared and field-tested as described in Materials and Methods. Beads prepared in this fashion resulted in a stable, continuous release of the odorant compounds; the release rate at 5 days post preparation remained high for almost all of the compounds, ranging from 67–95% of the initial amount released (Fig. 5). The only exception to this was hexanal, which decayed more rapidly than the other compounds, with only 28% of the original amount remaining at day 5 (Fig. 5).

**Figure 6:**
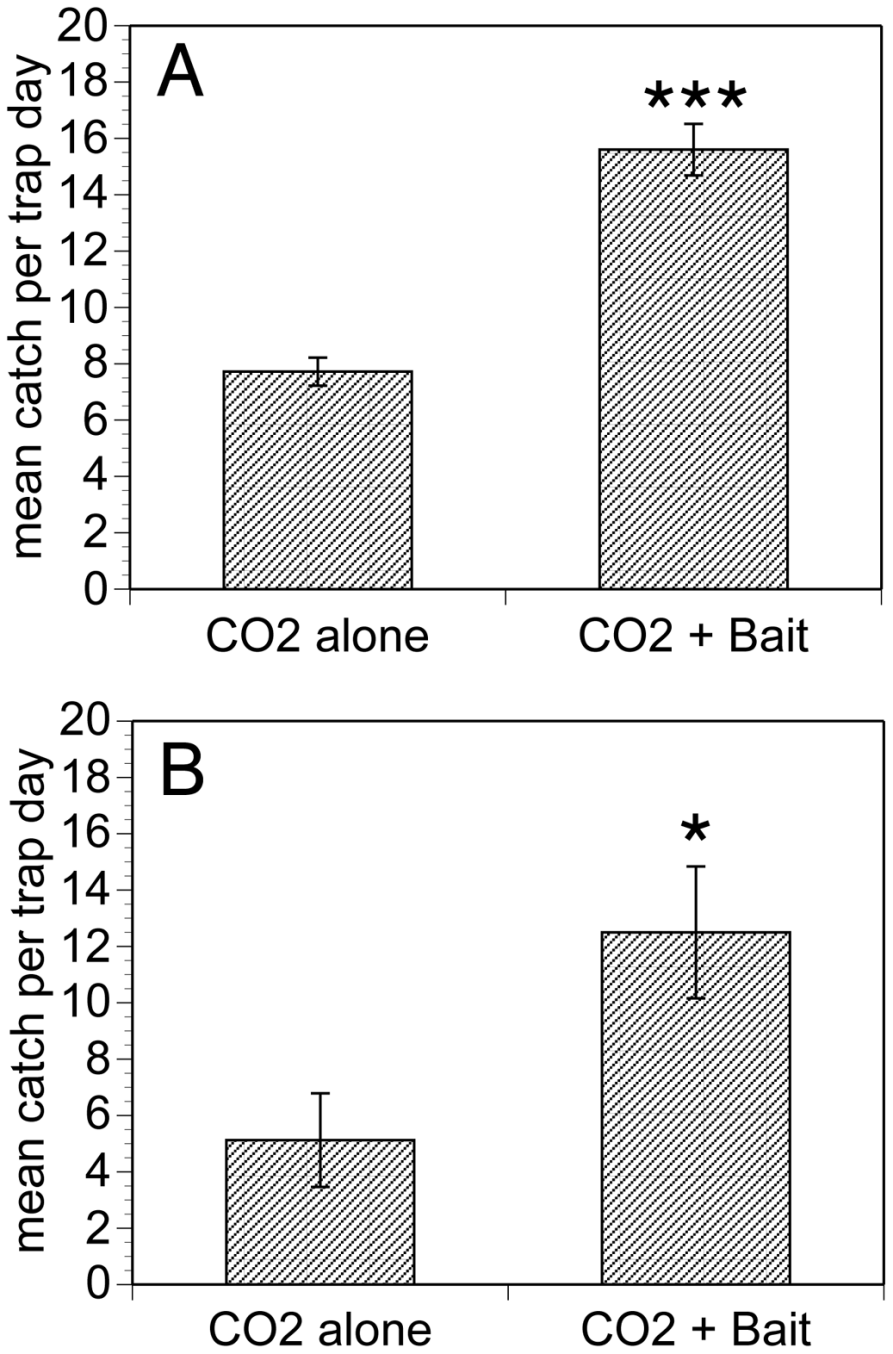
Field evaluation of baits containing attractive compounds. Panel A: Collection of *Simulium ochraceum s.l.* on optimized EWT platforms baited with CO_2_ only or CO_2_ + selected human odorants (bait). Odorants included in the bait: 1-octen-3-ol, 1-octenol, acetophenone, hexanal, and ammonium bicarbonate. Data were analyzed using a negative binomial regression model and significance determined using a Wald chi square test. In Panel A, *** indicates p<0.0001. Panel B: Collection of *Simulium damnosum s.l.* on optimized EWT platforms baited with CO_2_ only or CO_2_ + selected human odorants. Odorants included in the bait: hexanoic acid, heptanoic acid, octanoic acid nonanonic acid, acetophenone, decanal and ammonium bicarbonate. In Panel B, * indicates p<0.01. In each panel, bars indicate the mean collection per trap per day and error bars the standard error of the mean.

Pantyhose were loaded with the beads and ammonium bicarbonate and used as baits on optimized EWT platforms (e.g. Fig. 1, Panel G). Traps were baited with both CO_2_ and the attractant-impregnated beads, as previous studies had indicated that odor baits were not effective unless used in conjunction with CO_2_ [8], [9]. The CO_2_ for the traps was generated from a solution of baker's yeast and sugar, as previously described [8]. Traps baited with CO_2_ and the aroma bead baits containing the compounds found to be attractive for *S. ochraceum* s.l. in the Y tube assays (1-octen-3-ol, 1-octanol, acetophenone, hexanal, and ammonium bicarbonate) collected roughly twice the number of *S. ochraceum* s.l. in Mexico than did the traps baited with CO_2_ alone (Fig. 6, Panel A; p<0.001). Similarly, traps baited with the compounds found to be attractive for *S. damnosum* s.l. in the Y tube assays (hexanoic acid, heptanoic acid, octanoic acid, nonanoic acid, 1-decanal, acetophenone, and ammonium bicarbonate) and CO_2_ collected roughly three times as many *S. damnosum* s.l. in Burkina Faso as did traps baited with CO_2_ alone (Fig. 6, Panel B; p<0.01). Thus, the compounds identified as attractive in the EAG and binary choice behavioral assays were also attractive to the target species under field conditions.

## Discussion

Early field studies in Africa indicated that human sweat contains compounds that attract *Simulium damnosum s.l.*, the major African *O. volvulus* vector species group [22], [23]. Unidentified compounds in soiled clothing were effective in promoting host-seeking by several sibling species in this species complex, and, of the chemical and physical variables involved in host attraction, human odor emanating from sweat was uniquely required. The studies reported here provide insight into the particular compounds responsible for this attractive response.

We initially attempted to identify a suite of compounds that were common among three individuals, with the hypothesis that such common compounds would reflect a “common” human odor and thus allow us to establish a foundation for an attractive bait formulation. Surprisingly, we found a limited number of compounds that were shared among all three individuals. Just 29 compounds were found to be in common among the three individuals, of over 1200 compounds identified. This represents a smaller set of compounds than have been identified in other studies of human sweat components [24]–[26]. It is likely that many of the compounds that were not common among the individuals were derived from cosmetics and detergents, though the individuals enrolled in the study were asked to refrain from using deodorants and perfumed products during the time the collections were performed. It is also possible that some of the differences noted were due to differences in the skin microflora of the individuals, as bacterial metabolism of sweat has been previously shown to produce a number of attractive volatiles [16], [17]. Finally, some of the differences might be due to individual differences in metabolism. For example, previous studies have suggested an association between HLA genotype and attractiveness to malaria vectors that has been linked to differences in the profile of sweat components of individuals with different HLA haplotypes [27].

Interestingly, 1-octen-3-ol, an acknowledged host-seeking stimulant for a number of hematophagous Diptera [28], elicited a strong EAG response in both vector species, but was significantly attractive only to *S. ochraceum* in the Y-tube assay. 1-Octen-3-ol is a common component of human volatiles and sweat [29], [30]; however, field studies have shown this compound to be marginally attractive for several zoophilic North American black fly species when used singly [31], indicating this kairomone likely requires additional compounds such as CO_2_ to be optimally effective as a bait. The lack of attractiveness of 1-octen-3-ol to *S. damnosum s.l.* is therefore not unexpected, as members of this species group (including *S. sirbanum*) are mainly zoophilic and blood-feed on several host species other than humans, including cattle, donkeys, goats, sheep and dogs [32], [33].

In contrast to *S. damnosum s.l.*, the cytotype of *S. ochraceum s.l.* found in Mexico (cytotype A) is highly anthropophilic and prefers humans to other animal hosts [14]. The two species groups are also quite distant both geographically (Neotropic versus Afrotropic) and phylogenetically (subgenus *Psilopelmia* versus subgenus *Edwardsellum*) and as a result have likely evolved host-seeking behaviors independently within environments that differ greatly in phenology and the range of host species available as blood sources. *Simulium ochraceum* (cytotype A) is restricted in its flight range to the montane regions of Mexico and Guatemala where onchocerciasis was formerly endemic. *Simulium ochraceum s.l.* also consists of far fewer sibling taxa (n = 3) when compared to *S. damnosum s.l.* (≥50) indicating that gene flow resulting in sibling speciation has been less robust. Of the two species groups, *S. ochraceum* was stimulated and attracted by a larger, more chemically diverse number of human sweat components, suggesting that anthropophily as exemplified by this species may be associated with a relatively large number and variety of compounds in addition to CO_2_, thereby allowing *S. ochraceum* to exploit humans routinely as hosts and transmit *O. volvulus* in the discrete Mesoamerica foci.

*Simulium damnosum s.l.* females responded to several medium-chain carboxylic acids, particularly octanoic acid, a carboxylic acid that also evokes host-seeking behavior by other vector species in Africa. Carboxylic acids have been previously implicated as components contributing to human odor [24], [25] and several are attractive to important mosquito vectors. Octanoic acid is EAG-stimulatory and attractive to *Anopheles gambiae sensu stricto* [30], [34] and has been incorporated into a synthetic blend designed to attract various taxa in the *An. gambiae* species complex [11], [12]. This compound is also EAG-stimulatory and a significant host kairomone for *Culex quinquefasciatus*, an important lymphatic filariasis vector in Africa and the Indian subcontinent [35]. Decanal, a kairomone for zoophilic *S. damnosum s. l.,* also elicits a strong EAG response in *Cx. quinquefasciatus* [36] and is attractive to several mosquito vectors of Rift Valley Fever virus [37]. Decanal, a component of human odor [38], is also found on the skin of other hosts utilized by *S. damnosum s.l.* females, including cattle, goats and donkeys [30], [37].

Acetophenone is less obvious as a human host kairomone, but was EAG stimulatory and elicited attraction in both species. This compound has been reported as electro-stimulatory or behaviorally-attractive to several disparate hematophagous Diptera seeking a plant sugar meal. Acetophenone is a component of some floral nectaries and is a potent attractant to *Aedes aegypti* and *Cx. pipiens molestus* [39]. Kwon et al. [40] also noted that acetophenone was stimulatory to the labellar sensillum (S1) of *An*. *gambiae*. Nulliparous *S. damnosum s.l.* use plant sugars to fuel local host-seeking flight and sugar meals may also be essential for long-distance, migratory flight by savanna species in the *S. damnosum s.l* group. [41]; *S. ochraceum s.l.* females feed daily on plant sources and use floral carbohydrates as nutrients for flight and ovarian development [42]. However, because acetophenone also occurs as a volatile in human and bovine breath [43], [44] as a result of food digestion, it would likely be attractive for host-seeking hematophagus insects as well.

Acetophenone's status as a kairomone for both vector groups reinforces the concept of sensory parsimony, a phenomenon described only for several recently evolved blood-feeding Diptera. This process, i.e., dissimilar behavioral patterns cued by a single chemical compound, has been reported for *Stomoxys calcitrans* [45] and *Glossina* spp. [46] but never for *Simulium* spp. For example, *S. calcitrans* may use acetophenone resulting from rumen digestion to locate hosts for blood-feeding while several species of tsetse – *Glossina fuscipes*, *G*. *brevipalpis*, *G*. *pallidipes* (all obligate blood-feeders) - responded to 4-ethyl acetophenone as a chemo-attractant to locate *Lantana camara* for shelter. Our report of acetophenone as stimulatory to the Simuliidae when compared to those for the higher Diptera thus dates the phenomenon of sensory parsimony occurring ≈150 million years earlier in evolutionary time among blood-feeding flies [47].

Ammonium bicarbonate was also attractive to both *S. damnosum s.l.* and *S. ochraceum s.l.* Ammonia, a major degradation product of this inorganic salt, is found in both human sweat and breath and has been shown to be attractive to a wide range of blood-sucking insects [48]. When combined with lactic acid, ammonia also serves as a synergist to attract *Aedes aegypti*, the Yellow Fever mosquito, to human hosts.

In the current study, we concentrated upon compounds found to be in common among three different individuals, with the aim of identifying kairomones that were broadly attractive. The preliminary field trials demonstrated that the kairomones that we identified were attractive to both *S. ochraceum* s.l. and *S. damnosum* s.l. However, the number of flies captured using these compounds was relatively low, suggesting that the bait formulations will have to be optimized. Previous studies have demonstrated that the EWT, when baited with dirty clothing and CO_2_ collected numbers of vector flies that approached those obtained from human landing collections, suggesting that the appropriate mix of kairomones should be sufficient to allow the EWT's performance to be optimized to the point where it is a viable replacement for human landing collectors. Furthermore, it is well known that individuals differ in their attractiveness to hematophagous insects and that this difference can be traced to individual variation in odorant compounds [27], [49]. Thus, it is possible that the baits might be made more effective through the identification of those compounds that make certain individuals particularly attractive to the black fly vectors of *O. volvulus*. Comparative analysis of the GC-MS patterns of host volatiles collected from more and less attractive individuals, coupled with the EAG and behavioral assays described above could be used to identify such highly attractive compounds. Once optimized bait formulations have been developed it will be necessary to conduct extensive field trials to demonstrate that the EWTs will be able to replace human landing collectors for surveillance of *O. volvulus* transmission and to develop models to relate the collections obtained from the traps to those obtained from human landing collectors, thereby permitting one to estimate annual biting rates from the trap collections.

The EWT platforms have been designed to be inexpensive and simple to construct from materials that are commonly available in developing countries [8], [9]. The use of plastic aroma beads as vehicles for the attractive compounds is in keeping with this design goal. Aroma beads are easily available on-line and from craft stores, and are quite inexpensive (ca. $11 USD per kg). Thus, aroma bead based baits may be easily prepared locally by onchocerciasis elimination programs when needed, facilitating the adoption of the EWT trap as a surveillance toll to replace human landing collections in both Africa and Latin America.

In addition to their use for surveillance and evaluation of the effectiveness of mass drug campaigns, traps baited with highly attractive lures could also be important in removing host-seeking segments of the adult black fly population in endemic onchocerciasis settings. Stochastic models indicate that the addition of vector control to community directed drug treatment with Mectizan (ivermectin) can have a profound effect on *O. volvulus* populations by changing the threshold biting rate required to maintain the parasite [50]. By using the compounds described here as baits in conjunction with CO_2_, it is possible that the Esperanza Window Trap can not only increase the efficiency and safety of surveillance of *O. volvulus* transmission but may also assist in eliminating river blindness in Africa by reducing adult fly populations below parasite population transmission thresholds, particularly in hypo- and meso-endemic settings.
